# Early-life microbiota exposure and prevention of childhood asthma: immunological mechanisms and intervention perspectives

**DOI:** 10.3389/fped.2026.1872906

**Published:** 2026-07-30

**Authors:** Yan Wang, Jinyan Yu, Ying Wang, Yanlei Li

**Affiliations:** 1 Department of Respiratory and Critical Care Medicine, The Second Hospital of Jilin University, Changchun, China; 2 Department of Clinical Laboratory, The Second Hospital of Jilin University, Changchun, China

**Keywords:** asthma prevention, childhood asthma, gut microbiota, maternal, microbiota, training immunity

## Abstract

Asthma is one of the most prevalent chronic diseases among children, affecting approximately one in ten globally. Preventing asthma in the early life benefits individuals both during childhood and into adulthood. During the critical prenatal period, exposure to diverse maternal microbiota may enhance immune tolerance in fetal and neonatal stages. This exposure can train the immunity of innate immune cells through functional reprogramming of epigenetic mechanisms, potentially altering susceptibility to allergic diseases like asthma. Additionally, microbiota transmission during childbirth, close maternal-child contact, and breastfeeding influence offspring immunity. Furthermore, childhood exposure to a microbiota-rich environment, a diverse gut microbiota, and reduced viral infections are crucial for preventing asthma in children. Early-life exposure to diverse microbiota influences host cells via bacterial metabolites, soluble immune effectors, and other mediators, thereby shaping immune development. These microbial signals activate specific immune pathways and foster tolerance, changes that have been linked to a lower risk of allergic disease. Most evidence to date derives from observational human studies and animal models; consequently, mechanisms remain incompletely defined and standardized intervention strategies are lacking. Longitudinal human cohorts are therefore needed to delineate microbiota–host interaction mechanisms and to enable individualized, precision-based prevention of childhood asthma.

## 1 Introduction

Asthma is a major global non-communicable disease affecting both children and adults. By 2018, roughly 300 million people worldwide lived with asthma, and an additional 100 million people may be affected by 2025 (1, 2). In childhood, asthma commonly causes school absenteeism and reduced participation in physical activities, imposing substantial social and economic burdens on families and health care systems (3). Although many children experience symptomatic remission between school age and adolescence, resolution of clinical symptoms does not necessarily reflect the disappearance of airway hyperresponsiveness or airway inflammation (4). Consequently, effective prevention in early life could yield health benefits that extend into adulthood. Beyond genetic susceptibility, well-established environmental contributors to asthma development include nutrition, allergens, pollutants (particularly tobacco smoke), microbiota, and psychosocial factors. Current strategies for preventing childhood asthma concentrate on identifying modifiable environmental and host risk factors (5, 6). The role of the microbiota in the prevention of childhood asthma has received increasing attention.

**Figure 1:**
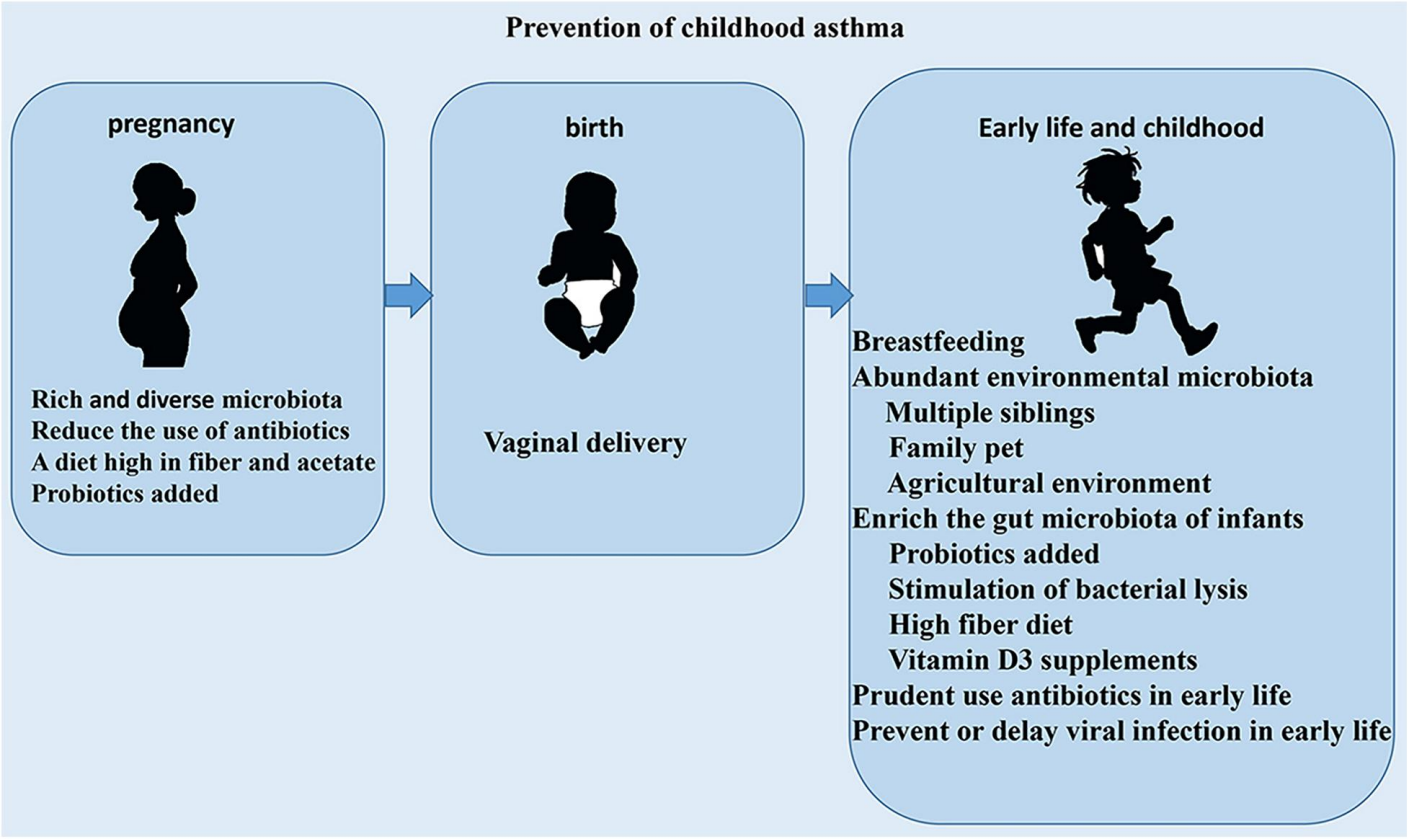
Summarizes the methods of microbiota enrichment to prevent childhood asthma. The methods described in the current research are summarized for each stage including pregnancy, delivery mode, and infant period. SCFAs: Short-chain fatty acids.

The term microbiota encompasses all bacteria, viruses, and eukaryotic organisms that inhabit or reside on the human body; The concept of exposure includes all exposures from the external environment as well as any endogenous biological systems (including those occurring within the body) (7). Over time, the original “hygiene hypothesis” has been refined into broader frameworks, which posit that early-life contact with diverse microbial communities lowers the risk of allergic diseases and asthma (8). Recent advances in multi-omics technologies and the precision medicine paradigm (9), now enable earlier identification of children at high risk for asthma by integrating host immune phenotyping, molecular biomarkers, and individualized microbiome profiles. Such integrated assessments provide a rational basis for targeted microbiome interventions—examples include specific probiotic strains, dietary modification, and controlled optimization of environmental microbial exposures (10). This review synthesizes current evidence linking the microbiome to childhood asthma prevention, emphasizing maternal microbial exposures, environmental and gut microbiota, and the roles of viruses and parasites; specific preventive strategies are summarized in Figure 1.

## 2 Methods

This narrative review synthesizes current evidence linking early-life microbiota exposure to the prevention of childhood asthma, with particular emphasis on underlying immune-regulatory mechanisms. We searched PubMed and the Web of Science Core Collection (SCIE, SSCI, and AHCI) for publications dated 2000–2026. The search combined subject terms and keywords including “childhood asthma”, “asthma prevention”, “microbiota”, “microbiota exposure”, “immune mechanism”, “gut microbiota”, “training immunity”, and “Th cells”. We included studies that met all of the following criteria: 1) research conducted in humans or in animal models; 2) focus on associations between prenatal, perinatal, or early-childhood microbiota exposure and asthma or allergic outcomes; and 3) experimental work addressing mechanistic pathways. Exclusion criteria were: secondary reviews of non-original data (except high-impact reviews), case reports, and conference abstracts. For screening, priority was given to longitudinal cohort studies, animal experiments with clearly defined mechanisms, and high-quality evidence published within the last five years. Although this review did not strictly follow the PRISMA systematic review protocol, its selection process referenced PRISMA principles where appropriate—specifically the clear definition of inclusion and exclusion criteria and the emphasis on methodologically rigorous studies.

## 3 Maternal microbiota and offspring asthma

Early-life microbiota exerts a lasting influence on human development and health (11, 12), including susceptibility to asthma (13, 14). Environmental exposures at critical windows (conception, birth, and the first postnatal year) can reprogram fetal and infant immune development and thereby modify disease risk (11, 15). In this context, maternal perinatal microbiota exposures are pivotal: by shaping fetal immune ontogeny, they help prevent allergic disorders in offspring (16, 17). Supporting this view, a longitudinal multi-omics study traced microbiome and metabolome co-development from late pregnancy through the first year of life and reported that horizontal gene transfer (HGT) from mother to infant alters the metabolic capacity of the infant gut microbiota, with downstream effects on immune development (18).

### 3.1 Prenatal exposure: intrauterine microbial signals and immune training

The long-standing view that the intrauterine environment is sterile has been challenged by molecular studies, the Deoxyribonucleic Acid (DNA) of bacteria has been detected in healthy amniotic fluid and the placenta, implying that host–microbe interactions may begin *in utero* (19, 20). During pregnancy, fetal and neonatal mucosal immunity can be influenced by microbial DNA, microbial metabolites, maternal microbial, and maternally derived antibodies (21–24). The microbiota exchange between mother and infant *in utero* may play an important role in infant prevention of allergies and asthma (25). Experimental work in animals shows that delayed microbial colonization impairs development of gut-associated lymphoid tissue, producing long-lasting immune dysregulation in mice (26, 27). Conversely, exposing pregnant germ-free mice to bacteria increases numbers of monocytes and type-3 innate lymphoid cells in the offspring's gut mucosa (28). Both human cohort and animal studies report that maternal antibiotic use during pregnancy alters the neonatal gut environment and is associated with higher offspring risk of allergic pulmonary inflammation and asthma (29–31). In addition, maternal stimulation with lipopolysaccharide (32), *Acinetobacter lwoffi* (33), or *Lactobacillus rhamnoseus* (34) during pregnancy reduces allergic responses and airway inflammation in offspring in animal models.

The “maternal-driven immune education” hypothesis proposes that contact with the mother during critical developmental windows enables offspring to develop optimal immune defenses against infection and inflammation (35). Immune training describes how exposure to infection or vaccination reprograms innate immune cells, inducing epigenetic memory that amplifies their responses upon subsequent challenge (36). It was found in murine experiments that metabolites derived from the maternal prenatal microbiota can be sensed by fetal bone marrow progenitors and exert persistent effects on the offspring's immune function (37). Comparative analyses of cord blood from farm and non-farm infants indicate prenatal immune conditioning: farm-exposed neonates exhibit elevated levels of microbial-perceived Toll-like receptors (TLRs) and some cytokines, changes that correlate with reduced asthma susceptibility (38, 39). Specific environmental microbiota (particularly those characteristic of rural or farming settings) can induce effective intrauterine immune training, accelerating neonatal immune maturation and counteracting a Th2-biased trajectory linked to asthma risk (40–42).

### 3.2 Microbiota transmission during delivery and breastfeeding

Mode of delivery is a key determinant of vertical microbiota transmission. In vaginal birth, maternal microbiota directly seeds the neonate's intestinal and mucosal surfaces, and subsequent close mother–infant contact and breastfeeding further broaden and sustain this transfer (43, 44). A human cohort study conducted in the Netherlands demonstrated that infants delivered via cesarean section harbored lower intestinal abundance of bifidobacteria and *Bacteroides*, and exhibited higher colonization prevalence of *Clostridioides difficile* compared with vaginally born infants (45). A Danish cohort reported an elevated risk of asthma among children delivered by cesarean section compared with those born vaginally (46). A cross-sectional study in China similarly identified an age-dependent association between cesarean delivery and both asthma occurrence and the timing of first wheeze in offspring (47). Moreover, breast milk through its resident microbes and oligosaccharides shapes the developing infant's gut microbiome and establishes functional links between beneficial microbes and immune regulation during the first months of life (48, 49). A maternal low-fiber diet during pregnancy and lactation may affect the establishment of the offspring's gut microbiota, which in turn diminishes the infant's resistance to respiratory tract infections (50). Antibiotic transfer through breast milk in mice impairs regulatory T cell (Treg) differentiation in co-cultured T cells, provoking dysregulated Th1 responses to bacterial challenge and intensifying allergic reactions (51). Breastfeeding is a crucial way to transfer the maternal microbiota to the infant, and it may extend the effects of the maternal-induced immune changes beyond the period of birth (52).

### 3.3 Immune mechanism: From TLR signaling to treg-mediated immune tolerance

Maternal exposure to an environment rich in microbiota upregulates of innate immune receptors (TLR2 and TLR4) expression in offspring (39, 53). Via pathogen-associated molecular patterns (PAMPs), microbiota activate pattern recognition receptors (especially TLRs) on innate immune cell surfaces, leading to the rapid onset of innate immune responses (54). TLRs play a key role in the regulation of innate cell function in asthma, and the inhibitory capacity of Tregs is indirectly or directly regulated by the TLR ligands through antigen-presenting cells(APCs) or responsive T cells (55). Dendritic cells (DCs) are the most potent APCs. Tolerogenic dendritic cells (tolDCs) enforce immune tolerance principally by suppressing effector T cell responses and promoting Tregs induction (56–58). The bacterial metabolite butyrate modulates epithelial inflammatory responses and antigen tolerance by inducing tolDCs and stimulating anti-inflammatory cytokine production (59). Likewise, intratracheal administration of the bacterial lysate OM-85 induces tolDCs and attenuates allergic airway inflammation in mice (60). Together, these findings link maternal microbiota exposure, TLR signaling, tolDCs-mediated tolerance, and Tregs as a coherent pathway influencing neonatal immunity.

**Figure 2:**
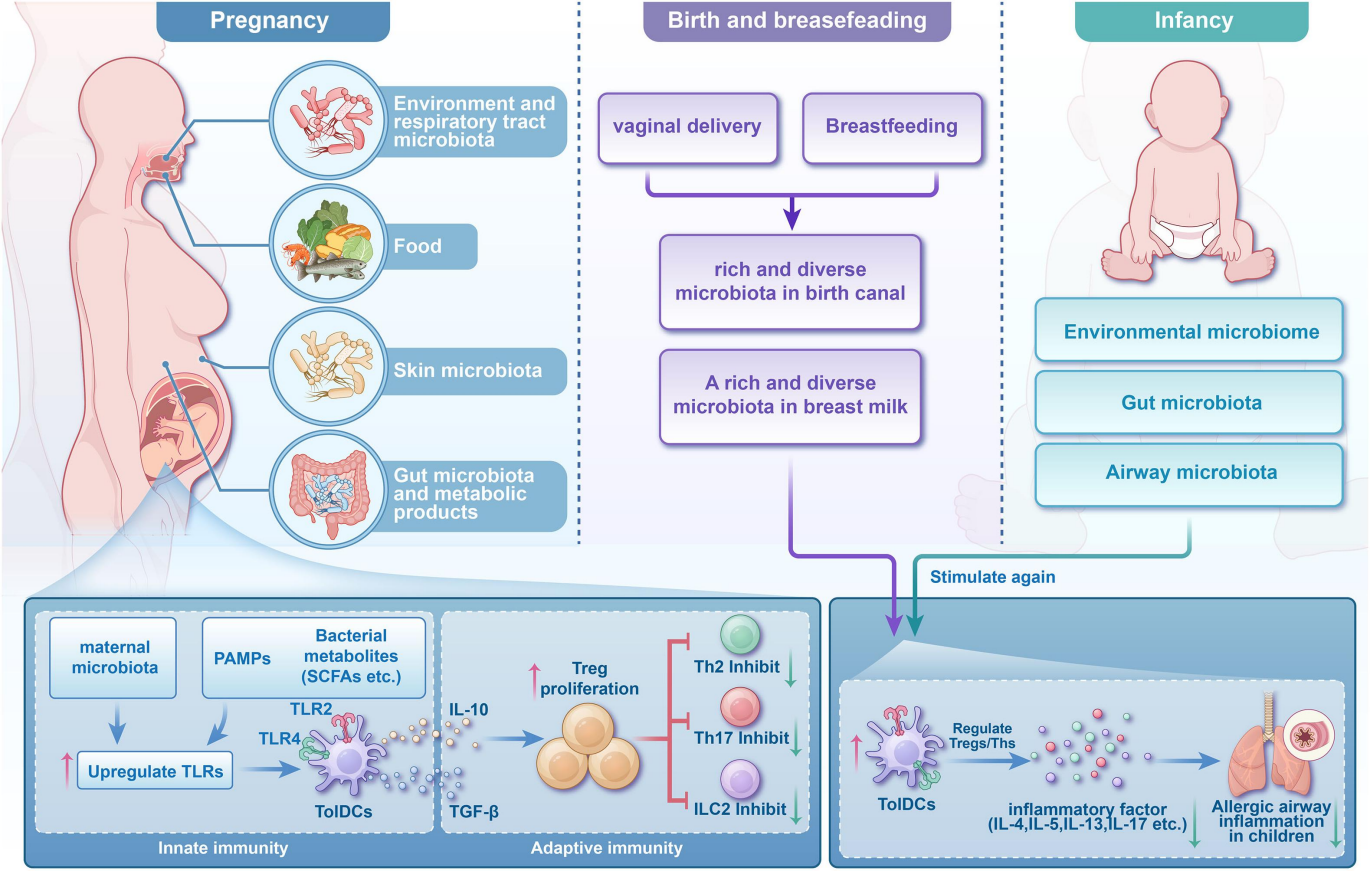
The immune mechanism of maternal microbiota exposure prevents asthma in the offspring. Maternal microbiota exposure (in rural or farm environments, exposure to specific microbiota) can stimulate the fetus, increasing the expression of TLR2 and TLR4 in umbilical cord blood. The PAMPs of microbiota are recognized by TLRs, which induce tolDC and secrete cytokines (such as IL-10, TGF-*β*), promoting Treg differentiation, inhibiting Th2, Th17 effector cells, ILC2 (the most abundant ILC subtype at the maternal-fetal interface), mast cells, etc., mediating immune tolerance; after birth, due to the upregulation of TLR2, the tolerance characteristics of tolDC can be amplified. When tolDCs encounters microbial stimulation again, it will produce higher levels of IL-10, while low levels of pro-inflammatory factors are secreted, inhibiting allergic airway inflammation in children. The above mechanisms collectively form a regulator*y* axis that proceeds from “maternal microbiota exposure → TLR/DC-mediated signal transmission → Treg expansion → suppression of Th2/Th17 responses and ILC2 inhibition → establishment of offspring immune tolerance”. TLR, Toll-like Receptor; PAMPs, pathogen-associated molecular patterns; tolDCs, tolerogenic dendritic cells; IL, Interleukin; TGF-*β*, transforming growth factor-*β*; Treg, Regulatory T cells; Th, T helper cell; ILC2, Innate lymphoid cells.

Tregs maintain adaptive immune homeostasis and suppress Th2-driven allergic inflammation (61, 62). Although Th2 cells were long considered the principal source of Th2 cytokines such as interleukin −13 (IL-13) and IL-5, recent work has identified type 2 innate lymphoid cells (ILC2s) as an additional, potent producer of these mediators (63, 64). ILC2 is the most abundant subtype of ILC at the human maternal-fetal interface during preterm and term gestations (65). Animal models indicate ILC2s contribute importantly to allergic disease pathogenesis (66). Tregs can inhibit Th2 and other immune cells involved in the inflammatory response (67), including ILC2 (68), mast cells, basophils, effector Th2/Th1/Th17 cells (69, 70). Maternal microbiota exposure modulates fetal immune development via the TLRs–DCs–Tregs-Ths axis, a form of microbial-mediated immune training that enhances offspring responsiveness to subsequent microbial challenges and lowers offspring asthma risk (see Chapter 3.1). Figure 2 summarizes the immune mechanisms by which maternal microbiota exposure protects offspring from asthma.The protective effect of the above-mentioned maternal microbiota exposure on the offspring's asthma, cannot fully explain all the differences in children's asthma risk. Other factors such as the external environmental microbiota directly encountered by children, as well as their complex interactions with urbanization and air pollution, also need to be considered.

## 4 Environmental microbiota and childhood asthma

Environmental microbiota are associated with allergic disease risk early in life by modulating immune responses at skin, airway, and gut mucosal surfaces and by influencing immune system development (71).

### 4.1 The evolution of the “hygiene hypothesis”

In 1989, David Strachan reported an inverse association between hay fever and the number of siblings and coined the “hygiene hypothesis” to explain rising allergy rates (72). He attributed this increase to demographic and lifestyle changes—smaller families, urbanization, modern habits, and increasingly sanitized environments. Building on Strachan's work, Graham Rook and colleagues advanced the “old friends hypothesis,” arguing that people in affluent urban settings increasingly lack exposure to organisms with which humans co-evolved; this loss predisposes to dysregulated inflammatory responses (73). The “biodiversity hypothesis” contact with diverse environmental microbes enriches host microbial communities (74), promotes balanced immune responses, and thereby reduces the risk of allergic and inflammatory disorders (75). Tracing this conceptual evolution—from the “hygiene hypothesis” to the “biodiversity hypothesis”—has progressively clarified the role of environmental microbiota in early immune maturation and inflammation control. Numerous studies have since confirmed a link between environmental microbial exposures and the development of childhood asthma.

### 4.2 The impact of environmental microbiota exposure on childhood asthma

Long-term contact with domestic animals in early life and living in environments rich in diverse bacteria and fungi, have been associated with a reduced risk of childhood asthma (76). Increased microbial biodiversity, particularly the presence of protective species, appears to dampen Th2 cell activation (77). Supporting this mechanism, an animal study found that isolator-reared piglets had a markedly Tregs population, farm-reared piglets exhibited a significantly higher Treg-to-effector ratio (78). An environmental assessment of urban children in the United States indicated that concurrent early-life exposure to elevated levels of certain allergens and bacteria may be protective (79). Within agricultural settings, despite a similar lifestyle and genetic background, children on dairy farms that use livestock for transportation had lower incidences of asthma and allergic diseases than children on large industrial farms, the innate immune cell phenotype and function are also significant differences (80). Similarly, two cross-sectional studies of pediatric allergic disease reported that farm-dwelling children encounter a broader diversity of environmental microorganisms and show lower prevalences of asthma and atopy compared with reference populations (81, 82). Overall, the reduced incidence of asthma observed in rural vs. urban children is correlated with an exposure to higher concentrations of environmental microbial communities (83).

The simple urban–rural dichotomy inadequately captures the heterogeneity of environmental microbial exposures. Rural settings are not uniformly protective: microbiota exposure profiles and asthma incidence vary across rural types—traditional farming villages, industrialized farms, pastoral communities, and suburban rural zones (84). Likewise, asthma risk differs substantially among urban neighborhoods, including older inner-city districts, newly developed high-rise residential areas, and urban fringe zones (85, 86). For example, a Danish cohort study found that among children raised in highly urbanized areas, greater exposure to urban green space was associated with a lower risk of developing asthma (86).

### 4.3 Impacts of socioeconomic factors and air pollution on environmental microbiota and childhood asthma

Socioeconomic status influences not only a family's ability to reside in neighborhoods with greater green space and cleaner air but also determines housing quality, parenting practices, nutritional status, access to medical care, and parents' work-related stress and time allocation (87–89). Moreover, environmental microbial exposure interacts intricately with air pollution. Outdoor pollutants (Particulate Matter 2.5, nitrogen dioxide) (90) and indoor contaminants(nitrogen dioxide and volatile organic compounds) (91), disrupt respiratory microenvironments, remodel local microbiota, and weaken or even reverse the protective benefits of early microbial exposure (92, 93). In some urban settings, heavy traffic-related pollution appears to blunt the immunoregulatory actions of beneficial microbes, may amplify airway inflammation and amplify airway inflammation (94, 95), which in turn raises childhood asthma risk (96). Consequently, increasing microbial diversity alone is not uniformly beneficial; the health outcome depends on the composition of the microbiota, exposure dose, background pollution, and individual genetic susceptibility acting together.

In conclusion, the pathways through which environmental microbiota influence childhood asthma remain incompletely understood and warrant further investigation. Rather than relying on simplistic urban–rural comparisons, future research should integrate microbiota exposure profiles with socioeconomic context, concurrent air pollution effects, and host immune characteristics to derive more precise, mechanism-informed prevention strategies.

## 5 Gut microbiota and childhood asthma

For many years, clinicians and researchers have argued that gut microbiota can be deleterious in critically ill patients by promoting systemic inflammation and facilitating infection (97). This concern stems in part from the risk that opportunistic translocation of intestinal microbes can pose to a compromised host (98). More recently, however, studies have demonstrated that the gut microbiota also performs essential functions for the host: synthesizing vitamins, aiding digestion, and modulating immune responses. These activities help maintain immune homeostasis, provide key signals for immune system maturation and function, support mucosal defenses, and may influence systemic immunity (99–101). Despite the clear links between altered microbial communities and disease, no consensus definition of “dysbiosis” exists; current usages tend to loosely equate microbial imbalance with a range of chronic conditions without precise, universally accepted criteria (102).

### 5.1 Early-life gut microbiota and risk of childhood asthma

The gut microbiota influences the risk of immune-mediated diseases, including allergic diseases and asthma (103). As mentioned earlier, early-life gut microbiota composition and function are shaped by maternal microbial exposure, mode of delivery, breastfeeding, dietary intake, and antibiotic use. In the postpartum period, progressive gut microbiota colonization parallels maturation of the innate and adaptive immune systems (104). Microbial seeding of mucosal surfaces during infancy can exert durable effects: it may promote tolerance to environmental antigens or, conversely, predispose individuals to later-life disorders including inflammatory bowel disease, allergies, and asthma (105–107). Lower bacterial diversity in early-life microbiota has been linked to subsequent allergic disease development (108). Consistent with this, the Canadian Healthy Infant Longitudinal Development (CHILD) study found reductions in both total bacterial abundance and fecal acetate levels among children at high risk for asthma (109). A longitudinal analysis from infancy through school age identified persistent differences in the relative abundance of specific gut bacterial genera in children with Immunoglobulin E(IgE)-mediated allergic disorders compared with healthy controls (110). A Copenhagen prospective birth cohort likewise reported an inverse association between gut bacterial diversity during the first year of life (1–12 months) and the risk of allergic sensitization (111). Early-life antibiotic exposure disrupts the developing gut microbiota, perturbs immune maturation, and has been associated with increased risk of childhood asthma (112). Conversely, a Canadian prospective population-based analysis attributed part of the recent decline in childhood asthma incidence to more judicious infant antibiotic prescribing (113, 114).

These observational and animal studies collectively indicate a strong link between early microbiota perturbation and asthma risk, but cannot fully exclude reverse causation or unmeasured confounding. To date, the causal relationship between reduced gut microbial diversity and childhood asthma unresolved.

### 5.2 Strategies for intervening in the gut microbiota

Given the critical role of the gut microbiota in immune function and the pathogenesis of allergic diseases, numerous therapeutic strategies have been developed to modulate the intestinal microbiome (115).

#### 5.2.1 Bacteria and probiotics

Numerous animal studies have investigated the role of gut microbiota in modulating airway inflammation. In several murine models, supplementation with *Bifidobacterium breve* and *Lactobacillus rhamnosus* produced anti-inflammatory effects comparable to those of budesonide, suggesting potential utility in chronic asthma management (116, 117). Similarly, administration of *Lactobacillus johnsonii* to wild-type mice attenuated Th2-driven airway inflammation (118) and expanded Treg populations (119). Other animal experiments have shown that bacterial extracts or specific gut microbiota can ameliorate ovalbumin-induced airway inflammation (109, 120). Consistent with these preclinical findings, cross-sectional surveys report that early-life exposure to bacteria commonly found on farms is associated with lower rates of childhood allergic disease (121)^.^ Despite this converging evidence from observational and preclinical work, large-scale randomized controlled trials in humans are still scarce.

Administration of probiotics to neonatal mice increased Treg production associated with transforming growth factor–*β* (TGF-*β*), which in turn suppressed allergic sensitization and airway disease (122). Cohort studies in humans likewise indicate that probiotics can enhance immune regulation and mitigate food allergies in children (123, 124). The evidence remains insufficient to recommend specific probiotic strains for the prevention of allergic diseases and asthma (125, 126), Contradictory data further temper enthusiasm (127). Thus, while mechanistic and preliminary clinical data are promising, it is premature to be overly optimistic about the routine clinical use of probiotics for preventing childhood asthma.

#### 5.2.2 Bacterial lysates

Bacterial lysates, derived from microbial species most implicated in human respiratory tract infections, are prepared as mechanically separated particulate fractions or chemically degraded lyophilized cells (128). Clinical studies indicate that bacterial lysates engage TLRs in both adults and children, interact with dendritic cells residing in the mucosa of the upper respiratory and gastrointestinal tracts, and promote Treg responses; collectively, these mechanisms correlate with reductions in respiratory infections and improvements in allergic disease symptoms (129–131). In a cohort of school-aged children with allergic asthma, oral administration of bacterial lysis products significantly decreased exacerbation rates (132). Although bacterial lysates therefore constitute a promising preventive strategy, the current clinical evidence remains insufficient to support routine use for asthma prevention; additional well-designed, high-quality clinical trials are required.

#### 5.2.3 SCFAs and vitamin D

Microbial metabolomics enables the qualitative and quantitative characterization of small-molecule metabolites generated by microbial communities, thereby clarifying the functions and biological roles of these compounds (133). The microbiota communicates with the host via multiple modalities, including bacterial metabolites (134). Among microbial products, short-chain fatty acids (SCFAs) such as butyrate and propionate are generated by gut bacteria during the metabolism of specific dietary substrates (135). Dietary fiber selectively promotes the expansion of SCFAs-producing taxa (136). the resulting SCFAs act as signaling molecules that modulate diverse host physiological processes, including Treg function (137, 138). Consistent with this mechanism, mice fed a high-fiber diet develop a distinct gut microbiota with elevated fecal acetate and increased Treg abundance and activity, which together lead to a marked attenuation of allergic airway disease (139). Human cohort studies have linked SCFAs concentrations to infant diet: infants with the highest fecal levels of butyric and propionic acids during the first year of life exhibited a marked reduction in atopic allergies and a lower likelihood of developing asthma between ages three and six (140). In a double-blind human trial, higher serum 25(OH)D levels correlated with greater abundance of beneficial bacteria and reduced loads of pathogenic bacteria (141). Consistent with these observations, animal and human studies indicate that vitamin D supplementation dampens inflammatory signaling in multiple cell types implicated in asthma pathogenesis (142).

#### 5.2.4 Parasite

Numerous animal studies report that helminth infection and helminth-derived proteins increase circulating Tregs and ameliorates allergic diseases, including asthma (143–146). Consistent with these findings, some human cohort studies suggest helminth infection is associated with greater gut microbial diversity and reduced asthma risk (147). Administration of recombinant Hookworm protein attenuates ovalbumin-induced airway hyperresponsiveness in mice via the induction of Tregs responses (148). However, the clinical literature is not uniform. A nested case-control study has found children with asthma were more likely to have helminthiasis, particularly with Trichiuris trichura (149). A meta-analysis similarly found that children infected with Toxocara spp. have a higher likelihood of asthma compared with uninfected peers (150). By contrast, a case–control study detected no statistically significant association between seropositivity to Toxocara sp. and asthma risk in children (151). Beyond these conflicting epidemiologic results, parasite infection therapy raises unresolved safety and ethical concerns. For these reasons, parasite-based interventions remain highly controversial.

Intervention strategies targeting the gut microbiota fall into three main categories: 1) administration of live bacteria (e.g., *Lactobacillus* and *Bifidobacterium*) or bacterial extracts (152, 153), 2) deliberate infection with certain parasite or use of recombinant parasite proteins; and 3) dietary modification. Although a robust body of evidence links early-life gut microbiota composition to subsequent childhood asthma risk, current data do not justify routine use of any specific probiotic strains, bacterial lysates, or parasite-based therapies for asthma prevention.

## 6 Reduced viral infection burden and childhood asthma prevention

Viral infections are major drivers of wheezing across pediatric age groups and are key precipitants of acute asthma exacerbations (154, 155). Among school-age children, respiratory viruses trigger roughly 85% of asthma exacerbations, with respiratory syncytial virus (RSV) and human rhinovirus (HRV) being the predominant pathogens (156, 157). A population-based prospective birth cohort identified an age-dependent link between RSV infection in infancy and subsequent childhood asthma (158). Responses of goblet and ciliated cells to RSV infection are blunted in patients with asthma (159). Severe RSV disease can also induce lasting alterations in the pulmonary environment, producing persistent lung dysfunction after resolution of the acute infection (160, 161), even leads to repeated wheezing (162). Similarly, bronchial epithelial cells from children predisposed to asthma display dysregulated interferon and inflammatory responses when infected with HRV (163). Consistent with this, Leigh and colleagues showed that HRV infection may promote features of airway remodeling in patients with asthma (164).

Nearly every child contracts RSV or HRV within the first two years of life, which raises a critical question: whether pre-existing developmental defects in the lungs or innate immune system drive increased asthma susceptibility (165)? Genome-wide association studies have identified that host genetic factors are related to the risk of virus-induced asthma. Risk alleles are concentrated at the 17q21 locus and within the cadherin-related family member 3 (CDHR3) gene; CDHR3 risk alleles are related to increased susceptibility to respiratory virus infections (166). Likewise, variants in ORMDL3 and GSDMB, genes located in the 17q12–q21 region on chromosome 17, are associated with asthma symptoms in children following HRV respiratory tract infections (167, 168). Epidemiological studies further show that birth timing relative to winter viral seasons carries a measurable risk of early childhood asthma (169), implying that delayed or avoided exposure to seasonal viruses in infancy can reduce that risk. Early phenotyping of host immune responses can facilitate the timely identification of children at high risk for asthma. Efforts to develop viral vaccines, screen novel antiviral agents, and create immunomodulatory therapies aimed at preventing, delaying, or more effectively treating severe infant viral infections therefore hold promise for lowering the incidence of childhood asthma.

## 7 Conclusion

Understanding of microbiota–host interactions in asthma has expanded markedly. Early-life exposure to a diverse and abundant microbiota appears to lower childhood asthma risk by shaping barrier tissues (skin, airway, gut mucosa) and by modulating systemic immune function. Maternal microbial exposure during pregnancy likewise influences fetal immune ontogeny via pathways involving TLRs, DCs, Tregs, ILC2s, and Th cells, thereby guiding maturation of offspring immune responses, increasing immune tolerance, and reducing postnatal asthma risk. These mechanistic insights point to promising prevention strategies—novel probiotics and gut consortia, targeted microbial therapies, biologics, helminth-based preparations, and viral vaccines among them. However, the bulk of supporting evidence remains observational or derived from animal models, and translational efforts confront significant obstacles. Key gaps include an incomplete understanding of microbial colonization dynamics, difficulty establishing causality, and uncertainty about optimal timing and dosing for interventions. Addressing these limitations will require well-designed longitudinal human studies to map the temporal evolution of microbe–host interactions and to resolve causal links. Such work is essential to develop individualized microbiota-based approaches and to provide the rigorous foundation needed for precision prevention of asthma.

## References

[1] Stern J Pier J Litonjua AA. Asthma epidemiology and risk factors. Semin Immunopathol. (2020) 42(1):5–15. 10.1007/s00281-020-00785-1 32020334

[2] Dharmage SC Perret JL Custovic A. Epidemiology of asthma in children and adults. Front Pediatr. (2019) 7:246. 10.3389/fped.2019.00246 31275909 PMC6591438

[3] Saglani S Fleming L Sonnappa S Bush A. Advances in the aetiology, management, and prevention of acute asthma attacks in children. Lancet Child Adolesc Health. (2019) 3(5):354–64. 10.1016/S2352-4642(19)30025-2 30902628

[4] Oluwole O Rennie DC Afanasieva A Lawson JA. Personal and early life factors associated with new-onset asthma, remission, and persistence of asthma in a 2-year follow-up of schoolchildren. J Asthma. (2021) 58(4):488–96. 10.1080/02770903.2019.1709865 31906746

[5] Polk BI Bacharier LB. Potential strategies and targets for the prevention of pediatric asthma. Immunol Allergy Clin North Am. (2019) 39(2):151–62. 10.1016/j.iac.2018.12.010 30954167

[6] Haahtela T Alenius H Lehtimäki J Sinkkonen A Fyhrquist N Hyöty H. Immunological resilience and biodiversity for prevention of allergic diseases and asthma. Allergy. (2021) 76(12):3613–26. 10.1111/all.14895 33959980

[7] Merra G Gualtieri P La Placa G Frank G Della Morte D De Lorenzo A. The relationship between exposome and microbiome. Microorganisms. (2024) 12(7):1386. 10.3390/microorganisms12071386 39065154 PMC11278511

[8] Perkin MR Strachan DP. The hygiene hypothesis for allergy—conception and evolution. Front Allergy. (2022) 3:1051368. 10.3389/falgy.2022.1051368 36506644 PMC9731379

[9] Matricardi PM Van Hage M Custovic A Korosec P Santos AF Valenta R. Molecular allergy diagnosis enabling personalized medicine. J Allergy Clin Immunol. (2025) 156(3):485–502. 10.1016/j.jaci.2025.01.014 39855360

[10] Galli SJ. Toward precision medicine and health: opportunities and challenges in allergic diseases. J Allergy Clin Immunol. (2016) 137(5):1289–300. 10.1016/j.jaci.2016.03.006 27155026 PMC4872702

[11] Stiemsma LT Michels KB. The role of the microbiome in the developmental origins of health and disease. Pediatrics. (2018) 141(4):e20172437. 10.1542/peds.2017-2437 29519955 PMC5869344

[12] DeVries A McCauley K Fadrosh D Fujimura KE Stern DA Lynch SV. Maternal prenatal immunity, neonatal trained immunity, and early airway microbiota shape childhood asthma development. Allergy. (2022) 77(12):3617–28. 10.1111/all.15442 35841380 PMC9712226

[13] Stiemsma LT Turvey SE. Asthma and the microbiome: defining the critical window in early life. Allergy Asthma Clin Immunol. (2017) 13:3. 10.1186/s13223-016-0173-6 28077947 PMC5217603

[14] Depner M Taft DH Kirjavainen PV Kalanetra KM Karvonen AM Peschel S. Maturation of the gut microbiome during the first year of life contributes to the protective farm effect on childhood asthma. Nat Med. (2020) 26(11):1766–75. 10.1038/s41591-020-1095-x 33139948

[15] Christoforidou Z Burt R Mulder I Gill BP Pluske J Kelly D. Development of immune cells in the intestinal Mucosa can be affected by intensive and extensive farm environments, and antibiotic use. Front Immunol. (2018) 9:1061. 10.3389/fimmu.2018.01061 29868021 PMC5964130

[16] McDonald B McCoy KD. Maternal microbiota in pregnancy and early life. Science. (2019) 365(6457):984–5. 10.1126/science.aay0618 31488675

[17] Kalbermatter C Fernandez Trigo N Christensen S Ganal-Vonarburg SC. Maternal Microbiota, early life colonization and breast milk drive immune development in the newborn. Front Immunol. (2021) 12:683022. 10.3389/fimmu.2021.683022 34054875 PMC8158941

[18] Vatanen T Jabbar KS Ruohtula T Honkanen J Avila-Pacheco J Siljander H. Mobile genetic elements from the maternal microbiome shape infant gut microbial assembly and metabolism. Cell. (2022) 185(26):4921–36.e15. 10.1016/j.cell.2022.11.023 36563663 PMC9869402

[19] Mishra A Lai GC Yao LJ Aung TT Shental N Rotter-Maskowitz A. Microbial exposure during early human development primes fetal immune cells. Cell. (2021) 184(13):3394–409.e20. 10.1016/j.cell.2021.04.039 34077752 PMC8240556

[20] Vidal MS Jr. Menon R. In utero priming of fetal immune activation: myths and mechanisms. J Reprod Immunol. (2023) 157:103922. 10.1016/j.jri.2023.103922 36913842 PMC10205680

[21] Tian M Li Q Zheng T Yang S Chen F Guan W. Maternal microbe-specific modulation of the offspring microbiome and development during pregnancy and lactation. Gut Microbes. (2023) 15(1):2206505. 10.1080/19490976.2023.2206505 37184203 PMC10187089

[22] Hornef MW Torow N. Layered immunity’ and the ‘neonatal window of opportunity’—timed succession of non-redundant phases to establish mucosal host-microbial homeostasis after birth. Immunology. (2020) 159(1):15–25. 10.1111/imm.13149 31777069 PMC6904599

[23] Gao Y Nanan R Macia L Tan J Sominsky L Quinn TP. The maternal gut microbiome during pregnancy and offspring allergy and asthma. J Allergy Clin Immunol. (2021) 148(3):669–78. 10.1016/j.jaci.2021.07.011 34310928

[24] Gao Y O’Hely M Quinn TP Ponsonby A-L Harrison LC Frøkiær H. Maternal gut microbiota during pregnancy and the composition of immune cells in infancy. Front Immunol. (2022) 13:986340. 10.3389/fimmu.2022.986340 36211431 PMC9535361

[25] Mueller NT Differding MK Sun H Wang J Levy S Deopujari V. Maternal bacterial engraftment in multiple body sites of cesarean section born neonates after vaginal seeding-a randomized controlled trial. mBio. (2023) 14(3):e0049123. 10.1128/mbio.00491-23 37074174 PMC10294643

[26] West CE Jenmalm MC Prescott SL. The gut microbiota and its role in the development of allergic disease: a wider perspective. Clin Exp Allergy. (2015) 45(1):43–53. 10.1111/cea.12332 24773202

[27] Wilburn AN McAlees JW Haslam DB Graspeuntner S Schmudde I Laumonnier Y. Delayed microbial maturation durably exacerbates Th17-driven asthma in mice. Am J Respir Cell Mol Biol. (2023) 68(5):498–510. 10.1165/rcmb.2022-0367OC 36622830 PMC10174167

[28] Gomez de Agüero M Ganal-Vonarburg SC Fuhrer T Rupp S Uchimura Y Li H. The maternal microbiota drives early postnatal innate immune development. Science. (2016) 351(6279):1296–302. 10.1126/science.aad2571 26989247

[29] Alhasan MM Cait AM Heimesaat MM Blaut M Klopfleisch R Wedel A. Antibiotic use during pregnancy increases offspring asthma severity in a dose-dependent manner. Allergy. (2020) 75(8):1979–90. 10.1111/all.14234 32064643

[30] Tai SK Lin YH Lin CH Lin MC. Antibiotic exposure during pregnancy increases risk for childhood atopic diseases: a nationwide cohort study. Eur J Med Res. (2024) 29(1):189. 10.1186/s40001-024-01793-9 38504329 PMC10953187

[31] Loewen K Monchka B Mahmud SM T Jong G Azad MB. Prenatal antibiotic exposure and childhood asthma: a population-based study. Eur Respir J. (2018) 52(1):1702070. 10.1183/13993003.02070-2017 29678946

[32] Reiprich M Rudzok S Schütze N Simon JC Lehmann I Trump S. Inhibition of endotoxin-induced perinatal asthma protection by pollutants in an experimental mouse model. Allergy. (2013) 68(4):481–9. 10.1111/all.12121 23409786

[33] Conrad ML Ferstl R Teich R Brand S Blümer N Yildirim AÖ. Maternal TLR signaling is required for prenatal asthma protection by the nonpathogenic microbe Acinetobacter lwoffii F78. J Exp Med. (2009) 206(13):2869–77. 10.1084/jem.20090845 19995952 PMC2806458

[34] Smout J Valentin C Delbauve S Pauwels J Köhler A Flamand V. Maternal Lactobacillus rhamnosus administration impacts neonatal CD4T-cell activation and prevents murine T helper 2-type allergic airways disease. Front Immunol. (2023) 13:1082648. 10.3389/fimmu.2022.1082648 36685549 PMC9847498

[35] Fernandes KA Lim AI. Maternal-driven immune education in offspring. Immunol Rev. (2024) 323(1):288–302. 10.1111/imr.13315 38445769

[36] Ochando J Mulder WJM Madsen JC Netea MG Duivenvoorden R. Trained immunity—basic concepts and contributions to immunopathology. Nat Rev Nephrol. (2023) 19(1):23–37. 10.1038/s41581-022-00633-5 36253509 PMC9575643

[37] Kimura I Miyamoto J Ohue-Kitano R Watanabe K Yamada T Onuki M. Maternal gut microbiota in pregnancy influences offspring metabolic phenotype in mice. Science. (2020) 367(6481):eaaw8429. 10.1126/science.aaw8429 32108090

[38] Pfefferle PI Büchele G Blümer N Roponen M Ege MJ Krauss-Etschmann S. Cord blood cytokines are modulated by maternal farming activities and consumption of farm dairy products during pregnancy: the PASTURE study. J Allergy Clin Immunol. (2010) 125(1):108–15.e1-3. 10.1016/j.jaci.2009.09.019 19969338

[39] Yu J Liu X Li Y Meng S Wu F Yan B. Maternal exposure to farming environment protects offspring against allergic diseases by modulating the neonatal TLR-tregs-th axis. Clin Transl Allergy. (2018) 8:34. 10.1186/s13601-018-0220-0 30140427 PMC6098605

[40] Martín-Cruz L Palomares O. Allergen-specific immunotherapy and trained immunity. Allergy. (2025) 80(3):677–89. 10.1111/all.16423 39641571 PMC11891420

[41] Von Mutius E Smits HH. Primary prevention of asthma: from risk and protective factors to targeted strategies for prevention. Lancet. (2020) 396(10254):854–66. 10.1016/S0140-6736(20)31861-4 32910907

[42] Holt PG Strickland DH Custovic A. Targeting maternal immune function during pregnancy for asthma prevention in offspring: harnessing the “farm effect"? J Allergy Clin Immunol. (2020) 146(2):270–2. 10.1016/j.jaci.2020.04.008 32333916

[43] Mitchell CM Mazzoni C Hogstrom L Bryant A Bergerat A Cher A. Delivery mode affects stability of early infant gut microbiota. Cell Rep Med. (2020) 1(9):100156. 10.1016/j.xcrm.2020.100156 33377127 PMC7762768

[44] Pan Z Walsh CJ Feehily C Nori SRC McAuliffe FM Cotter PD. Breastfeeding and the milk resistome shape the establishment and transmission of antibiotic resistance genes in the infant gut microbiome. Gut Microbes. (2025) 17(1):2541033. 10.1080/19490976.2025.2541033 40755225 PMC12323436

[45] Penders J Thijs C Vink C Stelma FF Snijders B Kummeling I. Factors influencing the composition of the intestinal microbiota in early infancy. Pediatrics. (2006) 118(2):511–21. 10.1542/peds.2005-2824 16882802

[46] Kristensen K Henriksen L. Cesarean section and disease associated with immune function. J Allergy Clin Immunol. (2016) 137(2):587–90. 10.1016/j.jaci.2015.07.040 26371844

[47] Lin J Yuan S Dong B Zhang J Zhang L Wu J. The associations of caesarean delivery with risk of wheezing diseases and changes of T cells in children. Front Immunol. (2021) 12:793762. 10.3389/fimmu.2021.793762 34970272 PMC8712489

[48] Kortman GAM Timmerman HM Schaafsma A Stoutjesdijk E Muskiet FAJ Nhien NV. Mothers’ breast milk composition and their respective infant’s gut Microbiota differ between five distinct rural and urban regions in Vietnam. Nutrients. (2023) 15(22):4802. 10.3390/nu15224802 PMC10675055 38004196

[49] Henrick BM Rodriguez L Lakshmikanth T Pou C Henckel E Arzoomand A. Bifidobacteria-mediated immune system imprinting early in life. Cell. (2021) 184(15):3884–98.e11. 10.1016/j.cell.2021.05.030 34143954

[50] Sikder MAA Rashid RB Ahmed T Sebina I Howard DR Ullah MA. Maternal diet modulates the infant microbiome and intestinal Flt3L necessary for dendritic cell development and immunity to respiratory infection. Immunity. (2023) 56(5):1098–114.e10. 10.1016/j.immuni.2023.03.002 37003256

[51] Zhang X Borbet TC Fallegger A Wipperman MF Blaser MJ Müller A. An antibiotic-impacted microbiota compromises the development of colonic regulatory T cells and predisposes to dysregulated immune responses. mBio. (2021) 12(1):e03335-20. 10.1128/mBio.03335-20 33531385 PMC7858066

[52] Dulfer EA Domínguez-Andrés J. Mechanisms involved in the transmission of trained immunity to offspring. J Allergy Clin Immunol. (2024) 154(5):1117–9. 10.1016/j.jaci.2024.06.006 38866208

[53] Ege MJ Bieli C Frei R Van Strien RT Riedler J Üblagger E. Prenatal farm exposure is related to the expression of receptors of the innate immunity and to atopic sensitization in school-age children. J Allergy Clin Immunol. (2006) 117(4):817–23. 10.1016/j.jaci.2005.12.1307 16630939

[54] Wenger M Grosse-Kathoefer S Kraiem A Pelamatti E Nunes N Pointner L. When the allergy alarm bells toll: the role of toll-like receptors in allergic diseases and treatment. Front Mol Biosci. (2023) 10:1204025. 10.3389/fmolb.2023.1204025 37426425 PMC10325731

[55] Zakeri A Yazdi FG. Toll-like receptor-mediated involvement of innate immune cells in asthma disease. Biochim Biophys Acta Gen Subj. (2017) 1861(1 Pt A):3270–7. 10.1016/j.bbagen.2016.08.009 27543676

[56] Takenaka MC Quintana FJ. Tolerogenic dendritic cells. Semin Immunopathol. (2017) 39(2):113–20. 10.1007/s00281-016-0587-8 27646959 PMC5296314

[57] Kang C Li X Liu P Liu Y Niu Y Zeng X. Tolerogenic dendritic cells and TLR4/IRAK4/NF-*κ*B signaling pathway in allergic rhinitis. Front Immunol. (2023) 14:1276512. 10.3389/fimmu.2023.1276512 37915574 PMC10616250

[58] Castenmiller C Keumatio-Doungtsop BC Van Ree R De Jong EC Van Kooyk Y. Tolerogenic immunotherapy: targeting DC surface receptors to induce antigen-specific tolerance. Front Immunol. (2021) 12:643240. 10.3389/fimmu.2021.643240 33679806 PMC7933040

[59] Salvi PS Cowles RA. Butyrate and the intestinal epithelium: modulation of proliferation and inflammation in homeostasis and disease. Cells. (2021) 10(7):1775. 10.3390/cells10071775 34359944 PMC8304699

[60] Pivniouk V Gimenes-Junior JA Ezeh P Michael A Pivniouk O Hahn S. Airway administration of OM-85, a bacterial lysate, blocks experimental asthma by targeting dendritic cells and the epithelium/IL-33/ILC2 axis. J Allergy Clin Immunol. (2022) 149(3):943–56. 10.1016/j.jaci.2021.09.013 34560105 PMC8901455

[61] Kearley J Barker JE Robinson DS Lloyd CM. Resolution of airway inflammation and hyperreactivity after *in vivo* transfer of CD4+CD25+regulatory T cells is interleukin 10 dependent. J Exp Med. (2005) 202(11):1539–47. 10.1084/jem.20051166 16314435 PMC1350743

[62] Kearley J Robinson DS Lloyd CM. CD4+CD25+regulatory T cells reverse established allergic airway inflammation and prevent airway remodeling. J Allergy Clin Immunol. (2008) 122(3):617–24.e6. 10.1016/j.jaci.2008.05.048 18672278 PMC3389733

[63] Li Y Wang Z Duan S Wang X Zhang Y Bachert C. TLR4(+)group 2 innate lymphoid cells contribute to persistent type 2 immunity in airway diseases. Nat Commun. (2025) 16(1):7108. 10.1038/s41467-025-62532-0 40753172 PMC12318100

[64] Kato A. Group 2 innate lymphoid cells in airway diseases. Chest. (2019) 156(1):141–9. 10.1016/j.chest.2019.04.101 31082387 PMC7118243

[65] Xu Y Romero R Miller D Silva P Panaitescu B Theis KR. Innate lymphoid cells at the human maternal-fetal interface in spontaneous preterm labor. Am J Reprod Immunol. (2018) 79(6):e12820. 10.1111/aji.12820 29457302 PMC5948134

[66] Barlow JL Bellosi A Hardman CS Drynan LF Wong SH Cruickshank JP. Innate IL-13-producing nuocytes arise during allergic lung inflammation and contribute to airways hyperreactivity. J Allergy Clin Immunol. (2012) 129(1):191–8.e1-4. 10.1016/j.jaci.2011.09.041 22079492

[67] Jheng MJ Kita H. Control of asthma and allergy by regulatory T cells. Int Arch Allergy Immunol. (2024) 186(1):87–102. 10.1159/000540407 39154634 PMC11729466

[68] Aron JL Akbari O. Regulatory T cells and type 2 innate lymphoid cell-dependent asthma. Allergy. (2017) 72(8):1148–55. 10.1111/all.13139 28160290

[69] Komlósi ZI Van De Veen W Kovács N Szűcs G Sokolowska M O'Mahony L. Cellular and molecular mechanisms of allergic asthma. Mol Aspects Med. (2022) 85:100995. 10.1016/j.mam.2021.100995 34364680

[70] Rigas D Lewis G Aron JL Wang B Banie H Sankaranarayanan I. Type 2 innate lymphoid cell suppression by regulatory T cells attenuates airway hyperreactivity and requires inducible T-cell costimulator-inducible T-cell costimulator ligand interaction. J Allergy Clin Immunol. (2017) 139(5):1468–77.e2. 10.1016/j.jaci.2016.08.034 27717665 PMC5378695

[71] Renz H Skevaki C. Early life microbial exposures and allergy risks: opportunities for prevention. Nat Rev Immunol. (2021) 21(3):177–91. 10.1038/s41577-020-00420-y 32918062

[72] Weiss ST. Eat dirt–the hygiene hypothesis and allergic diseases. N Engl J Med. (2002) 347(12):930–1. 10.1056/NEJMe020092 12239263

[73] Rook GA Lowry CA Raison CL. Microbial ‘old friends’, immunoregulation and stress resilience. Evol Med Public Health. (2013) 2013(1):46–64. 10.1093/emph/eot004 24481186 PMC3868387

[74] Von Hertzen L Hanski I Haahtela T. Natural immunity. Biodiversity loss and inflammatory diseases are two global megatrends that might be related. EMBO Rep. (2011) 12(11):1089–93. 10.1038/embor.2011.195 21979814 PMC3207110

[75] Haahtela T. A biodiversity hypothesis. Allergy. (2019) 74(8):1445–56. 10.1111/all.13763 30835837

[76] Frei R Heye K Roduit C. Environmental influences on childhood allergies and asthma—the farm effect. Pediatr Allergy Immunol. (2022) 33(6):e13807. 10.1111/pai.13807 35754122 PMC9327508

[77] Musso P Chiappini E Bernardini R. Human microbiome and allergic diseases in children: pathogenetic role and therapeutic options. Curr Pediatr Rev. (2020) 16(2):89–94. 10.2174/1573396315666191025110849 31654515

[78] Lewis MC Inman CF Patel D Schmidt B Mulder I Miller B. Direct experimental evidence that early-life farm environment influences regulation of immune responses. Pediatr Allergy Immunol. (2012) 23(3):265–9. 10.1111/j.1399-3038.2011.01258.x 22300455

[79] Lynch SV Wood RA Boushey H Bacharier LB Bloomberg GR Kattan M. Effects of early-life exposure to allergens and bacteria on recurrent wheeze and atopy in urban children. J Allergy Clin Immunol. (2014) 134(3):593–601.e12. 10.1016/j.jaci.2014.04.018 24908147 PMC4151305

[80] Stein MM Hrusch CL Gozdz J Igartua C Pivniouk V Murray SE. Innate immunity and asthma risk in amish and hutterite farm children. N Engl J Med. (2016) 375(5):411–21. 10.1056/NEJMoa1508749 27518660 PMC5137793

[81] Alfvén T Braun-Fahrländer C Brunekreef B Von Mutius E Riedler J Scheynius A. Allergic diseases and atopic sensitization in children related to farming and anthroposophic lifestyle–the PARSIFAL study. Allergy. (2006) 61(4):414–21. 10.1111/j.1398-9995.2005.00939.x 16512802

[82] Ege MJ Mayer M Normand A-C Genuneit J Cookson WOCM Braun-Fahrländer C. Exposure to environmental microorganisms and childhood asthma. N Engl J Med. (2011) 364(8):701–9. 10.1056/NEJMoa1007302 21345099

[83] Birzele LT Depner M Ege MJ Engel M Kublik S Bernau C. Environmental and mucosal microbiota and their role in childhood asthma. Allergy. (2017) 72(1):109–19. 10.1111/all.13002 27503830

[84] Pechlivanis S Von Mutius E. Effect of farming on asthma. Acta Med Acad. (2020) 49(2):144–55. 10.5644/ama2006-124.293 33189120

[85] Feleszko W Makrinioti H Nalej M Ooka T Zhu Z Sullivan AF. Early-life exposure to residential greenness and risk of asthma in a U.S. Bronchiolitis cohort. Allergy. (2024) 79(11):3036–46. 10.1111/all.16359 39429165 PMC11560528

[86] Coleman AT Teach SJ Sheehan WJ. Inner-City asthma in childhood. Immunol Allergy Clin North Am. (2019) 39(2):259–70. 10.1016/j.iac.2018.12.008 30954175

[87] Keller A Groot J Clippet-Jensen C Pinot de Moira A Pedersen M Sigsgaard T. Exposure to different residential indoor characteristics during childhood and asthma in adolescence: a latent class analysis of the Danish national birth cohort. Eur J Epidemiol. (2024) 39(1):51–65. 10.1007/s10654-023-01051-y 37865616 PMC10811114

[88] Zanobetti A Ryan PH Coull B Brokamp C Datta S Blossom J. Childhood asthma incidence, early and persistent wheeze, and neighborhood socioeconomic factors in the ECHO/CREW consortium. JAMA Pediatr. (2022) 176(8):759–67. 10.1001/jamapediatrics.2022.1446 35604671 PMC9127710

[89] Khan JR Lingam R Owens L Chen K Shanthikumar S Oo S. Social deprivation and spatial clustering of childhood asthma in Australia. Glob Health Res Policy. (2024) 9(1):22. 10.1186/s41256-024-00361-2 38910250 PMC11194868

[90] Klimkaite L Liveikis T Kaspute G Armalyte J Aldonyte R. Air pollution-associated shifts in the human airway microbiome and exposure-associated molecular events. Future Microbiol. (2023) 18:607–23. 10.2217/fmb-2022-0258 37477532

[91] Hulin M Caillaud D Annesi-Maesano I. Indoor air pollution and childhood asthma: variations between urban and rural areas. Indoor Air. (2010) 20(6):502–14. 10.1111/j.1600-0668.2010.00673.x 20846209

[92] Hamidou Soumana I Carlsten C. Air pollution and the respiratory microbiome. J Allergy Clin Immunol. (2021) 148(1):67–9. 10.1016/j.jaci.2021.05.013 34048853

[93] Vieceli T Tejada S Martinez-Reviejo R Pumarola T Schrenzel J Waterer GW. Impact of air pollution on respiratory microbiome: a narrative review. Intensive Crit Care Nurs. (2023) 74:103336. 10.1016/j.iccn.2022.103336 37440188

[94] Cheng SL Hedges M Keski-Rahkonen P Chatziioannou AC Scalbert A Chung KF. Multiomic signatures of traffic-related air pollution in London reveal potential short-term perturbations in gut microbiome-related pathways. Environ Sci Technol. (2024) 58(20):8771–82. 10.1021/acs.est.3c09148 38728551 PMC11112755

[95] Niemeier-Walsh C Ryan PH Meller J Ollberding NJ Adhikari A Reponen T. Exposure to traffic-related air pollution and bacterial diversity in the lower respiratory tract of children. PLoS One. (2021) 16(6):e0244341. 10.1371/journal.pone.0244341 34166366 PMC8224880

[96] Khreis H Kelly C Tate J Parslow R Lucas K Nieuwenhuijsen M. Exposure to traffic-related air pollution and risk of development of childhood asthma: a systematic review and meta-analysis. Environ Int. (2017) 100:1–31. 10.1016/j.envint.2016.11.012 27881237

[97] Clark JA Coopersmith CM. Intestinal crosstalk: a new paradigm for understanding the gut as the “motor” of critical illness. Shock. (2007) 28(4):384–93. 10.1097/shk.0b013e31805569df 17577136 PMC2084394

[98] Zhang CX Wang HY Chen TX. Interactions between intestinal Microflora/probiotics and the immune system. Biomed Res Int. (2019) 2019:6764919. 10.1155/2019/6764919 31828119 PMC6886316

[99] Schuijt TJ Lankelma JM Scicluna BP De Sousa E Melo F Roelofs JJTH De Boer JD. The gut microbiota plays a protective role in the host defence against pneumococcal pneumonia. Gut. (2016) 65(4):575–83. 10.1136/gutjnl-2015-309728 26511795 PMC4819612

[100] García-Montero C Fraile-Martínez O Gómez-Lahoz AM Pekarek L Castellanos AJ Noguerales-Fraguas F. Nutritional components in western diet versus Mediterranean diet at the gut Microbiota-immune system interplay. Implications for health and disease. Nutrients. (2021) 13(2):699. 10.3390/nu13020699 33671569 PMC7927055

[101] Wastyk HC Fragiadakis GK Perelman D Dahan D Merrill BD Yu FB. Gut-microbiota-targeted diets modulate human immune status. Cell. (2021) 184(16):4137–53.e14. 10.1016/j.cell.2021.06.019 34256014 PMC9020749

[102] Van Hul M Cani PD Petitfils C D Vos WM Tilg H El-Omar EM. What defines a healthy gut microbiome? Gut. (2024) 73(11):1893–908. 10.1136/gutjnl-2024-333378 39322314 PMC11503168

[103] Galazzo G Van Best N Bervoets L Dapaah IO Savelkoul PH Hornef MW. Development of the Microbiota and associations with birth mode, diet, and atopic disorders in a longitudinal analysis of stool samples, collected from infancy through early childhood. Gastroenterology. (2020) 158(6):1584–96. 10.1053/j.gastro.2020.01.024 31958431

[104] Koren O Konnikova L Brodin P Mysorekar IU Collado MC. The maternal gut microbiome in pregnancy: implications for the developing immune system. Nat Rev Gastroenterol Hepatol. (2024) 21(1):35–45. 10.1038/s41575-023-00864-2 38097774 PMC12635954

[105] Ames SR Lotoski LC Azad MB. Comparing early life nutritional sources and human milk feeding practices: personalized and dynamic nutrition supports infant gut microbiome development and immune system maturation. Gut Microbes. (2023) 15(1):2190305. 10.1080/19490976.2023.2190305 37055920 PMC10114993

[106] Ovaska M Tamminen M Lahdenperä M Vahtera J Rautava S Gonzales-Inca C. The role of early life factors and green living environment in the development of gut microbiota in infancy: population-based cohort study. Environ Int. (2024) 193:109093. 10.1016/j.envint.2024.109093 39490300

[107] West CE Rydén P Lundin D Engstrand L Tulic MK Prescott SL. Gut microbiome and innate immune response patterns in IgE-associated eczema. Clin Exp Allergy. (2015) 45(9):1419–29. 10.1111/cea.12566 25944283

[108] Zimmermann P Messina N Mohn WW Finlay BB Curtis N. Association between the intestinal microbiota and allergic sensitization, eczema, and asthma: a systematic review. J Allergy Clin Immunol. (2019) 143(2):467–85. 10.1016/j.jaci.2018.09.025 30600099

[109] Arrieta M-C Stiemsma LT Dimitriu PA Thorson L Russell S Yurist-Doutsch S. Early infancy microbial and metabolic alterations affect risk of childhood asthma. Sci Transl Med. (2015) 7(307):307ra152. 10.1126/scitranslmed.aab2271 26424567

[110] Simonyté Sjödin K Hammarström M-L Rydén P Sjödin A Hernell O Engstrand L. Temporal and long-term gut microbiota variation in allergic disease: a prospective study from infancy to school age. Allergy. (2019) 74(1):176–85. 10.1111/all.13485 29786876

[111] Bisgaard H Li N Bonnelykke K Chawes BLK Skov T Paludan-Müller G. Reduced diversity of the intestinal microbiota during infancy is associated with increased risk of allergic disease at school age. J Allergy Clin Immunol. (2011) 128(3):646–52.e1-5. 10.1016/j.jaci.2011.04.060 21782228

[112] Ahmadizar F Vijverberg SJH Arets HGM De Boer A Turner S Devereux G. Early life antibiotic use and the risk of asthma and asthma exacerbations in children. Pediatr Allergy Immunol. (2017) 28(5):430–7. 10.1111/pai.12725 28423467

[113] Patrick DM Sbihi H Dai DLY Al Mamun A Rasali D Rose C. Decreasing antibiotic use, the gut microbiota, and asthma incidence in children: evidence from population-based and prospective cohort studies. Lancet Respir Med. (2020) 8(11):1094–105. 10.1016/S2213-2600(20)30052-7 32220282

[114] Lee TY Petkau J Saatchi A Marra F Turvey SE Lishman H. Impact analysis of infant antibiotic exposure on the burden of asthma: a simulation modeling study. Front Allergy. (2024) 5:1491985. 10.3389/falgy.2024.1491985 39764437 PMC11701168

[115] Gagliardi A Totino V Cacciotti F Iebba V Neroni B Bonfiglio G. Rebuilding the gut Microbiota ecosystem. Int J Environ Res Public Health. (2018) 15(8):1679. 10.3390/ijerph15081679 30087270 PMC6121872

[116] Sagar S Morgan ME Chen S Vos AP Garssen J Van Bergenhenegouwen J. Bifidobacterium breve and Lactobacillus rhamnosus treatment is as effective as budesonide at reducing inflammation in a murine model for chronic asthma. Respir Res. (2014) 15(1):46. 10.1186/1465-9921-15-46 24735374 PMC4029990

[117] Spacova I Van Beeck W Seys S Devos F Vanoirbeek J Vanderleyden J. Lactobacillus rhamnosus probiotic prevents airway function deterioration and promotes gut microbiome resilience in a murine asthma model. Gut Microbes. (2020) 11(6):1729–44. 10.1080/19490976.2020.1766345 32522072 PMC7524350

[118] Fujimura KE Demoor T Rauch M Faruqi AA Jang S Johnson CC. House dust exposure mediates gut microbiome Lactobacillus enrichment and airway immune defense against allergens and virus infection. Proc Natl Acad Sci U S A. (2014) 111(2):805–10. 10.1073/pnas.1310750111 24344318 PMC3896155

[119] Fonseca W Lucey K Jang S Fujimura KE Rasky A Ting HA. Lactobacillus johnsonii supplementation attenuates respiratory viral infection via metabolic reprogramming and immune cell modulation. Mucosal Immunol. (2017) 10(6):1569–80. 10.1038/mi.2017.13 28295020 PMC5599307

[120] Hagner S Harb H Zhao M Stein K Holst O Ege MJ. Farm-derived gram-positive bacterium Staphylococcus sciuri W620 prevents asthma phenotype in HDM- and OVA-exposed mice. Allergy. (2013) 68(3):322–9. 10.1111/all.12094 23369007

[121] Riedler J Eder W Oberfeld G Schreuer M. Austrian Children living on a farm have less hay fever, asthma and allergic sensitization. Clin Exp Allergy. (2000) 30(2):194–200. 10.1046/j.1365-2222.2000.00799.x 10651771

[122] Feleszko W Jaworska J Rha R-D Steinhausen S Avagyan A Jaudszus A. Probiotic-induced suppression of allergic sensitization and airway inflammation is associated with an increase of T regulatory-dependent mechanisms in a murine model of asthma. Clin Exp Allergy. (2007) 37(4):498–505. 10.1111/j.1365-2222.2006.02629.x 17430345

[123] Santos SCD Konstantyner T Cocco RR. Effects of probiotics in the treatment of food hypersensitivity in children: a systematic review. Allergol Immunopathol (Madr). (2020) 48(1):95–104. 10.1016/j.aller.2019.04.009 31477401

[124] Dargahi N Johnson J Donkor O Vasiljevic T Apostolopoulos V. Immunomodulatory effects of probiotics: can they be used to treat allergies and autoimmune diseases? Maturitas. (2019) 119:25–38. 10.1016/j.maturitas.2018.11.002 30502748

[125] Moura JCV Moura ICG Gaspar GR Mendes GMS Faria BAV Jentzsch NS. The use of probiotics as a supplementary therapy in the treatment of patients with asthma: a pilot study and implications. Clinics. (2019) 74:e950. 10.6061/clinics/2019/e950 31411278 PMC6683305

[126] Wang HT Anvari S Anagnostou K. The role of probiotics in preventing allergic disease. Children. (2019) 6(2):24. 10.3390/children6020024 30764558 PMC6406271

[127] Quin C Estaki M Vollman DM Barnett JA Gill SK Gibson DL. Probiotic supplementation and associated infant gut microbiome and health: a cautionary retrospective clinical comparison. Sci Rep. (2018) 8(1):8283. 10.1038/s41598-018-26423-3 29844409 PMC5974413

[128] Suárez N Ferrara F Rial A Dee V Chabalgoity JA. Bacterial lysates as immunotherapies for respiratory infections: methods of preparation. Front Bioeng Biotechnol. (2020) 8:545. 10.3389/fbioe.2020.00545 32582669 PMC7289947

[129] Kearney SC Dziekiewicz M Feleszko W. Immunoregulatory and immunostimulatory responses of bacterial lysates in respiratory infections and asthma. Ann Allergy Asthma Immunol. (2015) 114(5):364–9. 10.1016/j.anai.2015.02.008 25752734

[130] de Boer GM Żółkiewicz J Strzelec KP Ruszczyński M Hendriks RW Braunstahl GJ. Bacterial lysate therapy for the prevention of wheezing episodes and asthma exacerbations: a systematic review and meta-analysis. Eur Respir Rev. (2020) 29(158):190175. 10.1183/16000617.0175-2019 33246991 PMC9488706

[131] Kaczynska A Klosinska M Janeczek K Zarobkiewicz M Emeryk A. Promising immunomodulatory effects of bacterial lysates in allergic diseases. Front Immunol. (2022) 13:907149. 10.3389/fimmu.2022.907149 35812388 PMC9257936

[132] Emeryk A Bartkowiak-Emeryk M Raus Z Braido F Ferlazzo G Melioli G. Mechanical bacterial lysate administration prevents exacerbation in allergic asthmatic children-the EOLIA study. Pediatr Allergy Immunol. (2018) 29(4):394–401. 10.1111/pai.12894 29575037

[133] Bauermeister A Mannochio-Russo H Costa-Lotufo LV Jarmusch AK Dorrestein PC. Mass spectrometry-based metabolomics in microbiome investigations. Nat Rev Microbiol. (2022) 20(3):143–60. 10.1038/s41579-021-00621-9 34552265 PMC9578303

[134] Fischbach MA Segre JA. Signaling in host-associated microbial communities. Cell. (2016) 164(6):1288–300. 10.1016/j.cell.2016.02.037 26967294 PMC4801507

[135] Clarke G Stilling RM Kennedy PJ Stanton C Cryan JF Dinan TG. Minireview: gut microbiota: the neglected endocrine organ. Mol Endocrinol. (2014) 28(8):1221–38. 10.1210/me.2014-1108 24892638 PMC5414803

[136] Zhao L Zhang F Ding X Wu G Lam YY Wang X. Gut bacteria selectively promoted by dietary fibers alleviate type 2 diabetes. Science. (2018) 359(6380):1151–6. 10.1126/science.aao5774 29590046

[137] Smith PM Howitt MR Panikov N Michaud M Gallini CA Bohlooly-Y M. The microbial metabolites, short-chain fatty acids, regulate colonic treg cell homeostasis. Science. (2013) 341(6145):569–73. 10.1126/science.1241165 23828891 PMC3807819

[138] Furusawa Y Obata Y Fukuda S Endo TA Nakato G Takahashi D. Commensal microbe-derived butyrate induces the differentiation of colonic regulatory T cells. Nature. (2013) 504(7480):446–50. 10.1038/nature12721 24226770

[139] Thorburn AN McKenzie CI Shen S Stanley D Macia L Mason LJ. Evidence that asthma is a developmental origin disease influenced by maternal diet and bacterial metabolites. Nat Commun. (2015) 6:7320. 10.1038/ncomms8320 26102221

[140] Roduit C Frei R Ferstl R Loeliger S Westermann P Rhyner C. High levels of butyrate and propionate in early life are associated with protection against atopy. Allergy. (2019) 74(4):799–809. 10.1111/all.13660 30390309

[141] Charoenngam N Shirvani A Kalajian TA Song A Holick MF. The effect of Various doses of oral vitamin D(3) supplementation on gut Microbiota in healthy adults: a randomized, double-blinded, dose-response study. Anticancer Res. (2020) 40(1):551–6. 10.21873/anticanres.13984 31892611

[142] Hall SC Agrawal DK. Vitamin D and bronchial asthma: an overview of data from the past 5 years. Clin Ther. (2017) 39(5):917–29. 10.1016/j.clinthera.2017.04.002 28449868 PMC5607643

[143] Elmehy DA Abdelhai DI Elkholy RA Elkelany MM Tahoon DM Elkholy RA. Immunoprotective inference of experimental chronic trichinella spiralis infection on house dust mites induced allergic airway remodeling. Acta Trop. (2021) 220:105934. 10.1016/j.actatropica.2021.105934 33895144

[144] Zaiss MM Rapin A Lebon L Dubey LK Mosconi I Sarter K. The intestinal Microbiota contributes to the ability of helminths to modulate allergic inflammation. Immunity. (2015) 43(5):998–1010. 10.1016/j.immuni.2015.09.012 26522986 PMC4658337

[145] Ruff WE Greiling TM Kriegel MA. Host-microbiota interactions in immune-mediated diseases. Nat Rev Microbiol. (2020) 18(9):521–38. 10.1038/s41579-020-0367-2 32457482

[146] Bohnacker S Troisi F De Los Reyes Jiménez M Esser-von Bieren J. What can parasites tell US about the pathogenesis and treatment of asthma and allergic diseases. Front Immunol. (2020) 11:2106. 10.3389/fimmu.2020.02106 33013887 PMC7516051

[147] Easton AV Quiñones M Vujkovic-Cvijin I Oliveira RG Kepha S Odiere MR. The impact of anthelmintic treatment on human gut Microbiota based on cross-sectional and pre- and postdeworming comparisons in western Kenya. mBio. (2019) 10(2):e00519–19. 10.1128/mBio.00519-19 31015324 PMC6479000

[148] Navarro S Pickering DA Ferreira IB Jones L Ryan S Troy S. Hookworm recombinant protein promotes regulatory T cell responses that suppress experimental asthma. Sci Transl Med. (2016) 8(362):362ra143. 10.1126/scitranslmed.aaf8807 27797959

[149] Senaratna CV Perera PK Arulkumaran S Abeysekara N Piyumanthi P Hamilton GS. Association of helminth infestation with childhood asthma: a nested case-control study. Int J Infect Dis. (2023) 128:272–7. 10.1016/j.ijid.2023.01.004 36632894

[150] Aghaei S Riahi SM Rostami A Mohammadzadeh I Javanian M Tohidi E. Toxocara spp. Infection and risk of childhood asthma: a systematic review and meta-analysis. Acta Trop. (2018) 182:298–304. 10.1016/j.actatropica.2018.03.022 29573999

[151] Cadore PS Zhang L Lemos LL Lorenzi C Telmo PL Dos Santos PC. Toxocariasis and childhood asthma: a case-control study. J Asthma. (2016) 53(6):601–6. 10.3109/02770903.2015.1064951 27104477

[152] Liévin-Le Moal V Servin AL. Anti-infective activities of lactobacillus strains in the human intestinal microbiota: from probiotics to gastrointestinal anti-infectious biotherapeutic agents. Clin Microbiol Rev. (2014) 27(2):167–99. 10.1128/CMR.00080-13 24696432 PMC3993101

[153] Kawahara T Makizaki Y Oikawa Y Tanaka Y Maeda A Shimakawa M. Oral administration of Bifidobacterium bifidum G9-1 alleviates rotavirus gastroenteritis through regulation of intestinal homeostasis by inducing mucosal protective factors. PLoS One. (2017) 12(3):e0173979. 10.1371/journal.pone.0173979 28346473 PMC5367788

[154] Gern JE Rosenthal LA Sorkness RL Lemanske RF Jr. Effects of viral respiratory infections on lung development and childhood asthma. J Allergy Clin Immunol. (2005) 115(4):668–74. 10.1016/j.jaci.2005.01.057 15805982 PMC7119046

[155] Aldakheel FM. Allergic diseases: a comprehensive review on risk factors, immunological mechanisms, link with COVID-19, potential treatments, and role of allergen bioinformatics. Int J Environ Res Public Health. (2021) 18(22):12105. 10.3390/ijerph182212105 34831860 PMC8622387

[156] Makrinioti H Hasegawa K Lakoumentas J Xepapadaki P Tsolia M Castro-Rodriguez JA. The role of respiratory syncytial virus- and rhinovirus-induced bronchiolitis in recurrent wheeze and asthma-A systematic review and meta-analysis. Pediatr Allergy Immunol. (2022) 33(3):e13741. 10.1111/pai.13741 35338734

[157] Ding Q Xu L Zhu Y Xu B Chen X Duan Y. Comparison of clinical features of acute lower respiratory tract infections in infants with RSV/HRV infection, and incidences of subsequent wheezing or asthma in childhood. BMC Infect Dis. (2020) 20(1):387. 10.1186/s12879-020-05094-4 32473625 PMC7260463

[158] Rosas-Salazar C Chirkova T Gebretsadik T Chappell JD Peebles RS Dupont WD. Respiratory syncytial virus infection during infancy and asthma during childhood in the USA (INSPIRE): a population-based, prospective birth cohort study. Lancet. (2023) 401(10389):1669–80. 10.1016/S0140-6736(23)00811-5 37086744 PMC10367596

[159] Gay ACA Banchero M Carpaij O Kole TM Apperloo L Van Gosliga D. Airway epithelial cell response to RSV is mostly impaired in goblet and multiciliated cells in asthma. Thorax. (2024) 79(9):811–21. 10.1136/thorax-2023-220230 38373824 PMC11347251

[160] Hon KL Leung AKC Wong AHC Dudi A Leung KKY. Respiratory syncytial virus is the most common causative agent of viral bronchiolitis in young children. An updated review. Curr Pediatr Rev. (2023) 19(2):139–49. 10.2174/1573396318666220810161945 35950255

[161] Carroll KN Gebretsadik T Escobar GJ Wu P Li SX Walsh EM. Respiratory syncytial virus immunoprophylaxis in high-risk infants and development of childhood asthma. J Allergy Clin Immunol. (2017) 139(1):66–71.e3. 10.1016/j.jaci.2016.01.055 27212083 PMC5074917

[162] Jartti T Bønnelykke K Elenius V Feleszko W. Role of viruses in asthma. Semin Immunopathol. (2020) 42(1):61–74. 10.1007/s00281-020-00781-5 31989228 PMC7066101

[163] Doni Jayavelu N Benson B Dela Cruz PC Powell WT Rich LM Vanderwall ER. Bronchial epithelial transcriptome reveals dysregulated interferon and inflammatory responses to rhinovirus in exacerbation-prone pediatric asthma. JCI Insight. (2025) 10(24):e197711. 10.1172/jci.insight.197711 PMC12890530 41217821

[164] Shariff S Shelfoon C Holden NS Traves SL Wiehler S Kooi C. Human rhinovirus infection of epithelial cells modulates airway smooth muscle migration. Am J Respir Cell Mol Biol. (2017) 56(6):796–803. 10.1165/rcmb.2016-0252OC 28257236

[165] Holgate ST Wenzel S Postma DS Weiss ST Renz H Sly PD. Asthma. Nat Rev Dis Primers. (2015) 1(1):15025. 10.1038/nrdp.2015.25 27189668 PMC7096989

[166] Forsström V Toivonen L Homil K Waris M Pedersen C-ET Bønnelykke K. Association of asthma risk alleles with acute respiratory tract infections and wheezing illnesses in young children. J Infect Dis. (2023) 228(8):990–8. 10.1093/infdis/jiad075 36967681 PMC10582910

[167] Çalışkan M Bochkov YA Kreiner-Møller E Bønnelykke K Stein MM Du G. Rhinovirus wheezing illness and genetic risk of childhood-onset asthma. N Engl J Med. (2013) 368(15):1398–407. 10.1056/NEJMoa1211592 23534543 PMC3755952

[168] Schoettler N Gebretsadik T Singh S Gress L Mendonça EA Snyder BM. Genotypes in the 17q12-q21 asthma risk locus and early-life viral wheezing illnesses. Pediatr Allergy Immunol. (2025) 36(8):e70165. 10.1111/pai.70165 40755347 PMC12319653

[169] Almqvist C Ekberg S Rhedin S Fang F Fall T Lundholm C. Season of birth, childhood asthma and allergy in a nationwide cohort-mediation through lower respiratory infections. Clin Exp Allergy. (2020) 50(2):222–30. 10.1111/cea.13542 31782836

